# Severe Withdrawal Symptoms and Pseudohyperchloremia Induced by Bromvalerylurea: A Case Report

**DOI:** 10.7759/cureus.78645

**Published:** 2025-02-06

**Authors:** Tatsuya Watanabe, Hiroyuki Shiga, Yusuke Tsutsumi, Noriyuki Kato, Yoshiaki Inoue

**Affiliations:** 1 Department of Emergency Medicine, National Hospital Organization (NHO) Mito Medical Center, Ibaraki, JPN; 2 Department of Emergency and Critical Care Medicine, University of Tsukuba Hospital, Tsukuba, JPN; 3 Department of Psychiatry, National Hospital Organization (NHO) Mito Medical Center, Ibaraki, JPN; 4 Department of Neurological Surgery, National Hospital Organization (NHO) Mito Medical Center, Ibaraki, JPN

**Keywords:** bromide, hypnotics and sedatives, pseudohyperchloremia, substance abuse, withdrawal symptom

## Abstract

Bromvalerylurea is a sedative-hypnotic agent commonly available in over-the-counter (OTC) medications in Japan and other regions. Although widely used, its prolonged and unsupervised use poses significant risks of dependency and withdrawal. Abrupt discontinuation of bromvalerylurea can lead to severe withdrawal symptoms, including agitation, hallucinations, and seizures. However, standardized management protocols for bromvalerylurea withdrawal have not been established.

A 19-year-old male with a suspected history of attention deficit hyperactivity disorder presented with severe agitation, hallucinations, and a generalized tonic-clonic seizure after abruptly discontinuing the sedative containing bromvalerylurea. He had been using an OTC sedative for nine months, progressively increasing his daily intake from three to 24 tablets. Upon admission, his vital signs were stable, but his serum chloride levels exceeded the measurable upper limit due to pseudohyperchloremia caused by bromide, a metabolite of bromvalerylurea. Initial management included oral diazepam 5 mg three times daily, which was tapered over nine days, leading to a resolution of withdrawal symptoms and normalization of chloride levels. The patient was transferred to a psychiatric facility on day 17 for substance use disorder treatment. Drug assays revealed undetectable bromvalerylurea levels by admission, while allylisopropylacetylurea levels declined by day 8.

This case underscores the severe withdrawal risks associated with bromvalerylurea, demonstrating the effectiveness of diazepam in stabilizing symptoms during withdrawal. Additionally, pseudohyperchloremia caused by bromide metabolism highlights the importance of recognizing this laboratory finding to avoid unnecessary interventions. With bromvalerylurea products being phased out as OTC medications due to safety concerns, efforts must focus on raising awareness about their risks and preventing misuse through online sales.

## Introduction

Bromvalerylurea is a sedative-hypnotic agent available in over-the-counter (OTC) medications in certain regions, including Japan. Although not widely recognized outside of certain areas, bromvalerylurea was discovered in the early 20^th^ century and has historically been used as a sleep aid and sedative for mild anxiety and insomnia [1]. Despite its long history, its OTC availability creates an overlooked dependency risk when taken long-term [2]. In Japan, bromvalerylurea is commonly included in OTC sedative-hypnotic formulations at doses of 250 mg per three tablets, though higher doses may be consumed in cases of misuse or dependence [3].

Bromvalerylurea enhances γ-aminobutyric acid A (GABA_A_) receptor activity leading to sedative effects [1]. Chronic use induces tolerance and dependency, and abrupt discontinuation can cause severe withdrawal symptoms, including agitation, hallucinations, and seizures, similar to benzodiazepines and barbiturates [4]. Furthermore, bromvalerylurea undergoes metabolism to bromide, a compound with a prolonged half-life that can accumulate in the body, resulting in pseudohyperchloremia, a laboratory abnormality that may lead to misdiagnosis [2,5].

The abrupt discontinuation of bromvalerylurea disrupts GABAergic modulation, leading to a hyperexcitable state in the central nervous system [1,4]. This dysregulation may result in severe neuropsychiatric symptoms, including agitation, vivid hallucinations, and seizures [6-9]. Despite the seriousness of these effects, reports on bromvalerylurea withdrawal are extremely rare, with most existing publications confined to Japanese literature.

This case highlights a rare instance of severe bromvalerylurea withdrawal, presenting with psychotic symptoms, seizures, and pseudohyperchloremia, successfully managed with diazepam. It aims to raise awareness among clinicians about the clinical and laboratory features of bromvalerylurea dependency, emphasizing its potential for severe withdrawal complications.

## Case presentation

A 19-year-old male with a history of prolonged OTC sedative use presented with agitation, vivid hallucinations, and a generalized tonic-clonic seizure following the abrupt discontinuation of bromvalerylurea. Laboratory findings revealed severe pseudohyperchloremia, which gradually resolved with diazepam tapering and supportive care.

Although he was suspected of having attention deficit hyperactivity disorder, he had never been formally diagnosed by a specialist.

Nine months before admission, he started using an OTC sedative containing bromvalerylurea (250 mg), allylisopropylacetylurea (150 mg), and diphenhydramine (25 mg) per three tablets, progressively increasing his daily intake from three to 24 tablets without medical supervision. One week before admission, he abruptly stopped taking the sedative.

The night before admission, he experienced intense auditory and visual hallucinations, including hearing "loud music" and seeing "large spiders." He became highly agitated, repeatedly turning lights on and off and irrationally claiming that "the house was flooded." 

On the day of admission, he developed severe anxiety and a three-minute generalized tonic-clonic seizure at home, leading to emergency transport via ambulance to the hospital. On arrival, he was disoriented but responsive. His vital signs were as follows: Glasgow Coma Scale score of E4V4M6, blood pressure of 147/98 mmHg, pulse rate of 112 beats/min, respiratory rate of 12 breaths/min, oxygen saturation (SpO_2_) of 97% (room air), and body temperature of 37.0℃. He exhibited no focal neurological deficits, and his pupils were bilaterally reactive at 4 mm. His seizure had already spontaneously resolved prior to arrival, he remained agitated, disoriented, and displayed pressured speech. Despite his agitation, he was able to provide a partial history, stating that he had stopped taking his sedative because he was concerned about his future: "I feel like I can't go on like this. I won't be able to live normally." Additional history was obtained from his mother at the bedside, who confirmed his recent behavioral changes and progressive increase in sedative use over the past months.

The patient's serum electrolytes were measured using ion-selective electrode (ISE) assays and were as follows: sodium, 142 mmol/L; potassium, 3.7 mmol/L; and chloride, which exceeded the measurable upper limit of 150 mmol/L (reference range: 98-108 mmol/L) likely due to bromide accumulation resulting from bromvalerylurea metabolism. Arterial blood gas analysis on room air yielded the following values: pH, 7.472; bicarbonate (HCO_3_^-^), 19.3 mmol/L; partial pressure of oxygen (PaO_2_), 90.7 mmHg; partial pressure of carbon dioxide (PaCO_2_), 27.0 mmHg; base excess, -2.8 mmol/L; and lactate, 2.9 mmol/L. The complete blood count and liver and renal function tests were within normal limits. A head CT scan showed no intracranial abnormalities. Although the ingested OTC sedative contained multiple active ingredients, bromvalerylurea was suspected as the primary cause of withdrawal symptoms due to its dependency-inducing properties.

**Figure 1:**
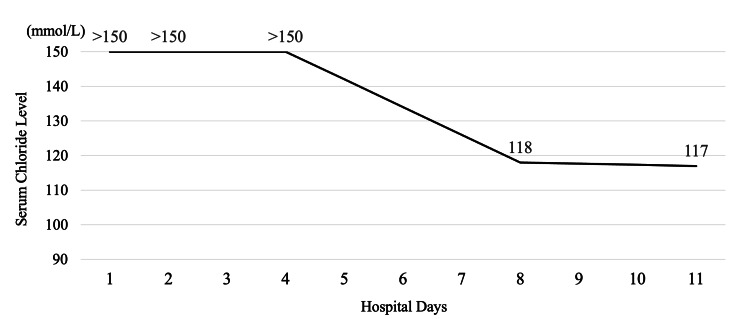
Changes in serum chloride levels during hospitalization The graph illustrates the changes in serum chloride levels measured during the patient’s hospitalization. On days 1, 2, and 4, serum chloride values exceeded the upper detection limit of 150 mmol/L using ion-selective electrode analysis and were recorded as >150 mmol/L. By day 8, the chloride concentration dropped to 118 mmol/L, and further decreased to 117 mmol/L on day 11, reflecting the gradual clearance of bromide from the patient’s system.

To prevent further seizures, oral diazepam 5 mg three times daily was initiated. Concurrently, a psychiatric consultation was obtained and interventions such as cognitive behavioral therapy and psychoeducation on substance use were initiated. Additionally, arrangements were made for transfer to a specialized rehabilitation facility. His agitation and confusion gradually improved within a few days, and he did not experience recurrent seizures or hallucinations. On day 2, electroencephalography (EEG) showed sleep wave patterns consistent with stage III sleep. On day 3, brain MRI showed no significant abnormalities. His chloride levels remained above 150 mmol/L until day 4, then gradually declined to 118 mmol/L by day 8 (Figure 1), coinciding with symptom improvement.

Diazepam was tapered by 5 mg every three days and discontinued on day 9. EEG on day 11 was within normal limits. On day 17, he was transferred to a psychiatric facility for substance use disorder treatment. Drug assays revealed undetectable bromvalerylurea levels, while allylisopropylacetylurea levels declined from 4.1 µg/mL on admission to undetectable by day 8 (Table 1).

**Table 1:** Analytical results of bromvalerylurea and allylisopropylacetylurea Liquid chromatography-mass spectrometry was used for analysis. ND: Not detected

Analyte	Day 1	Day 2	Day 4	Day 8	Day 11
Bromvalerylurea (μg/mL)	ND	ND	ND	ND	ND
Allylisopropylacetylurea (μg/mL)	4.1	1.5	0.1	ND	ND

## Discussion

Bromvalerylurea's OTC availability creates a clinical blind spot as patients and clinicians may underestimate its risks. Prolonged, unsupervised use can lead to severe withdrawal. This case highlights the need to raise clinician awareness of its dependency potential and establish structured withdrawal strategies. The patient’s OTC medication contained bromvalerylurea, allylisopropylacetylurea, and diphenhydramine. While allylisopropylacetylurea and diphenhydramine affect the central nervous system, they are not recognized as dependency-inducing agents [7]. Bromvalerylurea, however, has a well-documented potential for dependency, making it the most likely cause of the observed withdrawal symptoms [7]. Although several case reports describing bromvalerylurea withdrawal symptoms are available in Japanese literature (Table 2) [6-9], there is no established standard for withdrawal management strategies. Furthermore, English-language case reports remain exceedingly rare, emphasizing the need for increased international awareness of this issue.

**Table 2:** Summary of previous reports of bromvalerylurea withdrawal ISE: Ion-selective electrode; NA: Not available

Study	Age (year)/sex	Dosage (mg/day)	Daily use duration	Withdrawal onset time (days)	Symptoms	Chloride level on admission (mEql/L)	Chloride measurement method	Treatment for withdrawal	Complications
Our case	19/F	2000	9 months	7	Hallucination, seizure, agitation	More than 150	ISE assay	Benzodiazepine	None
Inoue, 2001 [6]	49/M	500-833	19 years	5	Hallucination, euphoria	106	NA	Levomepromazine	None
Inoue, 2001 [6]	35/F	1000-2000	11 years	5	Hallucination, agitation	107	NA	Diazepam, carbamazepine	None
Doi et al., 1998 [7]	39/F	1000	15 months	2	Hallucination, tremor	193	ISE assay	Haloperidol	None
Sano and Sakai, 2001 [8]	30/F	1000-2000	7 years	3	Hallucination, seizure	NA	NA	Sodium valproate	None
Tsuchida and Nishii, 2006 [9]	50s/F	1000-1500	5 years	0.5	Agitation	142	ISE assay	Diazepam	None

Chronic use of bromvalerylurea can lead to profound physiological and psychological adaptations, likely involving GABA_A_ receptors [1]. Such adaptations may result in dependency, as demonstrated by the patient’s severe withdrawal symptoms like agitation, hallucinations, and seizures after abrupt cessation. These symptoms likely stem from the brain’s response to the sudden loss of GABAergic modulation, leading to an overstimulated nervous system that may cause life-threatening symptoms [1,4,6]. We selected diazepam, a long-acting benzodiazepine, for managing this case due to its efficacy in stabilizing symptoms during GABAergic sedative withdrawal [1,4,9]. Although bromvalerylurea’s exact interaction with GABA_A_ receptors remains unclear, diazepam’s ability to maintain stable GABAergic transmission by countering excitatory rebound observed in GABAergic withdrawal made it a practical choice. A structured tapering regimen, such as reducing the dose by 5 mg every three days, may serve as a feasible strategy for managing similar cases. This structured tapering approach likely contributed to symptom resolution, allowing for a safe transfer to a psychiatric facility and demonstrating the effectiveness of diazepam in the complex scenario. 

In addition to diazepam, liaison psychiatry played a crucial role in this patient’s comprehensive management. Given the severity of withdrawal symptoms, psychiatric intervention was necessary for behavioral stabilization and long-term addiction support [10]. The psychiatrist provided cognitive-behavioral therapy and psychoeducation regarding substance use, facilitating the transition to a specialized addiction treatment facility. As in this case, early psychiatric consultation and timely transfer to a specialized psychiatric facility are vital to ensuring continuity of care and preventing relapse into drug abuse.

Another significant aspect of this case is the occurrence of pseudohyperchloremia, a misleading laboratory finding likely caused by bromvalerylurea use. Pseudohyperchloremia presents as elevated chloride levels on laboratory tests, with normal serum sodium and pH levels [2]. Although bromvalerylurea was undetectable in this patient’s blood, we attribute the pseudohyperchloremia to bromide, bromvalerylurea’s metabolite. Bromvalerylurea’s short half-life, approximately 2.5 hours [3,11], contributed to its rapid clearance from the bloodstream after discontinuation. In contrast, allylisopropylacetylurea, a co-ingested compound in this case, has a longer half-life of approximately 14 hours [12], explaining why it remained detectable in blood samples while bromvalerylurea did not. Although bromvaleryurea clears rapidly, its metabolite, bromide has a prolonged half-life of approximately 10-12 days, leading to its accumulation in the bloodstream and tissues [2]. Bromide’s chemical similarity causes interference in chloride measurement assays, resulting in falsely elevated chloride levels [2,5,7,9]. ISE assays are now the standard method for measuring electrolytes and were used in this case. However, previous studies have demonstrated that both ISE and non-ISE assays are affected by bromide to varying degrees [13]. Generally, a serum bromide level of 5 mEq/L increases chloride ion level by 20 mEq/L when measured via ISE assays [14]. This effect can last for several weeks, as bromide gradually clears from the body, depending on individual metabolism and renal function. The variability in chloride levels observed in previous reports, despite bromvalerylurea ingestion, may be attributed to differences in laboratory assay methods. However, as several previous reports do not specify the measurement methods used, the lack of standardization complicates direct comparisons (Table 2). Aside from bromvalerylurea, several other medications also contain bromide, including pyridostigmine bromide and rocuronium bromide [13]. Recognizing pseudohyperchloremia as a marker of bromide intake is essential for accurate diagnosis and effective treatment. Proper management involves discontinuing bromide intake and ensuring adequate hydration to promote bromide excretion [2,6,8]. Understanding this condition allows clinicians to avoid unnecessary interventions and to tailor treatment appropriately. 

As seen in this case, bromvalerylurea products are being phased out as OTC medications in Japan due to their high dependency potential and low safety profile, including pseudo-electrolyte abnormalities. However, there is an ongoing risk of these products being sold through online platforms, underscoring the need for enhanced regulatory measures and public awareness. Future research should focus on establishing standardized withdrawal management protocols and improving diagnostic approaches for bromide-related pseudohyperchloremia.

## Conclusions

This case highlights the withdrawal risks of OTC bromvalerylurea, as demonstrated by the patient’s severe neuropsychiatric symptoms, including agitation, hallucinations, and seizures following abrupt discontinuation. Pseudohyperchloremia, caused by bromide accumulation from bromvalerylurea metabolism, was a key diagnostic finding, underscoring the need for clinicians to recognize this laboratory abnormality to avoid misdiagnosis. Benzodiazepine tapering was likely effective in stabilizing withdrawal symptoms, while psychiatric intervention provided essential support for long-term management. These findings highlight the importance of structured pharmacological management in the acute phase, alongside psychiatric care to prevent relapse and ensure sustained recovery. With bromvalerylurea being phased out as an OTC medication, awareness of its risks remains crucial. Future efforts should focus on optimizing withdrawal management and improving recognition of bromide-related pseudohyperchloremia to prevent misdiagnosis and unnecessary interventions.
